# Antibiotic resistance and clonal diversity of invasive *Staphylococcus aureus* in the rural Ashanti Region, Ghana

**DOI:** 10.1186/s12879-016-2048-3

**Published:** 2016-11-29

**Authors:** Denise Dekker, Manuel Wolters, Eva Mertens, Kennedy Gyau Boahen, Ralf Krumkamp, Daniel Eibach, Norbert G. Schwarz, Yaw Adu-Sarkodie, Holger Rohde, Martin Christner, Florian Marks, Nimako Sarpong, Jürgen May

**Affiliations:** 1 Bernhard Nocht Institute for Tropical Medicine (BNITM), Research Group Infectious Disease Epidemiology, Bernhard-Nocht-Str. 74, D-20359 Hamburg, Germany; 2 German Centre for Infection Research (DZIF), Hamburg-Borstel-Lübeck, Hamburg, Germany; 3 University Medical Centre Hamburg-Eppendorf (UKE), Martinistr.52, D-20246 Hamburg, Germany; 4 Kumasi Centre for Collaborative Research in Tropical Medicine (KCCR), Kumasi, Ghana; 5 Kwame Nkrumah University of Science and Technology (KNUST), Kumasi, Ghana; 6 International Vaccine Institute (IVI), Seoul, Republic of Korea

**Keywords:** *S. aureus*, Rural ghana, *Spa* typing, Antibiotic resistance, Panton-Valentine leukocidin

## Abstract

**Background:**

*Staphylococcus aureus* is among the most common pathogens isolated from blood cultures in Ghana; yet the epidemiology of blood infections in rural settings is poorly described. This study aims to investigate antimicrobial susceptibility and clonal diversity of *S. aureus* causing bloodstream infections in two hospitals in the Ashanti Region, Ghana.

**Methods:**

Blood cultures were performed for all febrile patients (≥37.5 °C) on hospital admission. Antibiotic susceptibility testing for *S. aureus* isolates was carried out by the VITEK 2 system. Multiplex polymerase chain reaction (PCR) was used to detect *S. aureus*-specific *nuc* gene, Panton-Valentine leukocidin (PVL), and methicillin-resistant *S. aureus* (MRSA)-specific *mecA* and *mecC* genes. The population structure of *S. aureus* was assessed by *spa* typing.

**Results:**

In total, 9,834 blood samples were cultured, out of which 0.6% (*n* = 56) were positive for *S. aureus.* Multidrug resistance (MDR) was detected in 35.7% (*n* = 20) of the *S. aureus* strains, of which one was a MRSA. The highest rate of antibiotic resistance was seen for commonly available antibiotics, including penicillin (*n* = 55; 98.2%), tetracycline (*n* = 32; 57.1%) and trimethoprim/sulfamethoxazole (*n* = 26; 46.4%). Of all *S. aureus* strains, 75.0% (*n* = 42) carried the PVL-encoding genes. We found 25 different *spa* types with t355 (*n* = 11; 19.6%), t314 (*n* = 8; 14.3%), t084 (*n* = 8; 14.3%) and t311 (*n* = 5; 8.9%) being predominant.

**Conclusion:**

The study exhibited an alarmingly large level of antibiotic resistance to locally available antibiotics. The frequency of genetically diverse and PVL-positive methicillin-sensitive *S. aureus* (MSSA) was high and could represent a reservoir for the emergence of virulent PVL-positive MRSA clones.

## Background

Globally, *Staphylococcus aureus* is responsible for a variety of human infections including skin diseases, but also causing bacteraemia and sepsis [1]. In Ghana, *S. aureus* is the most frequently isolated pathogen from clinical specimens from hospitalized patients and ranks second among clinical isolates from outpatients [2]. Data from Ghanaian urban tertiary care hospitals have shown low antibiotic resistance and low rates of methicillin-resistant *S. aureus* (MRSA) [3]. In fact, low prevalence of invasive MRSA has been reported from several major towns in different African countries [4]. In contrast, a common attribute of *S. aureus* strains found in African communities appears to be the carriage of Panton-Valentine leukocidin (PVL), which is found at much higher rates than elsewhere [3, 5, 6]. There is evidence that PVL-positive isolates are more frequently detected in clinical isolates compared to asymptomatic colonization [7]. PVL is a cytotoxin encoded by the two genes *lukS*-*PV* and *lukF*-*PV* and thought to be associated with increased disease severity [8, 9], although its role in disease pathogenesis remains controversial [10, 11]. Studies have shown that some virulence factors such as PVL are frequently associated with certain genotypes [12]. In several studies *S. aureus* isolates from Ghana from different clinical samples and from nasal carriage have been characterized. In particular for rural settings, only few studies have investigated the clonal structure of isolated strains from blood cultures of febrile patients. However, these investigations are essential to develop and establish infection control strategies.

The objective of this study was to investigate antibiotic susceptibility, clonal diversity, and the occurrence of PVL in invasive *S. aureus* from blood cultures in the rural Ashanti Region, Ghana.

## Methods

### Study site, study population and ethical considerations

The study was conducted at two rural hospitals in the Ashanti Region in Ghana: the St. Michael’s Hospital (SMH) in Pramso in the Bosomtwe district and the Agogo Presbyterian Hospital (APH), situated in the Asante Akim North municipality.

Patients of all age groups, admitted to the hospitals with a tympanic temperature ≥ 37.5 °C or a history of fever in the last 24 h as well as neonates (aged ≤ 28 days) with suspected neonatal sepsis, were eligible for enrolment in this study. Excluded were patients with surgical or dermatological conditions. The study was conducted between May 2007 and August 2012, with different sampling periods at each hospital.

### Laboratory procedures

On admission, patient’s blood was drawn for blood culture. Small volumes of blood (1–3 ml) were inoculated into Becton Dickinson (BD) BACTEC® Peds Plus Medium and 8–10 ml of blood were inoculated into BD BACTEC® Plus Aerobic/F (Becton Dickinson, USA). Cultures were processed using a BACTEC® 9050 blood culture system (Becton Dickinson, USA) according to manufacturer’s instructions. For positive blood cultures, aspirated blood culture fluid was Gram stained for preliminary identification and inoculated on Columbia blood-, chocolate-, and MacConkey agar (all Oxoid, Basingstoke, UK). The plates were incubated at 37 °C for 18–24 h. Staphylococci isolates were presumptively identified by catalase positivity, free coagulase production (lyophilized rabbit plasma, bioMerieux, Marcy l’Etoile, France), and agglutination in the Staphaurex™ Plus test (Oxoid).

All bacterial strains were sent to Germany on dry ice for further analyses. Species identification of all *Staphylococcus* isolates was confirmed by MALDI-TOF MS (Bruker UK Limited, England) and by PCR detection of the *S. aureus*-specific *nuc* gene [13]. Antibiotic susceptibility testing was performed using the VITEK 2 system (AST 603 cards, bioMerieux, France) for penicillin, oxacillin, gentamicin, ciprofloxacin, moxifloxacin, erythromycin, clindamycin, linezolid, teicoplanin, vancomycin, tetracycline, tigecycline, fosfomycin, fusidic acid, rifampicin and trimethoprim/sulfamethoxazole. Breakpoints were applied according to the 2015 European Committee on Antimicrobial Susceptibility Testing (EUCAST) guidelines (http://www.eucast.org). Multidrug resistance (MDR) was defined as resistance to at least three groups of antibiotics or being an MRSA [14].

### DNA extraction and molecular typing

DNA was extracted using the automated QIAsymphony SP/AS instruments (QIAGEN, Germany). A multiplex PCR was used targeting the genes encoding PVL (*lukS*-*PV* and *lukF*-*PV*) and *mecA* and *mecC*, as described previously [13].

Sequence-based typing of the hypervariable region of *S. aureus* protein A (*spa*-typing) was performed as described by Harmsen and colleagues [15]. *Spa* types were assigned using the Ridom StaphType software version 2.2.1 (Ridom GmbH, Würzburg, Germany). Cluster analysis of *spa* typing data was performed by application of the integrated Based Upon Repeat Patterns (BURP) algorithm as described elsewhere [16]. The associated MLST-based sequence types or MLST-CCs were allocated by the Ridom SpaServer (http://spaserver.ridom.de), retrieved from the literature [1, 3, 12, 17, 18], or derived from closely related *spa*-types.

### Statistical analysis

Descriptive statistics were performed. Dichotomous variables were described using frequencies and their proportion. Continuous variables were described using the median along with the interquartile range (IQR). All analyses were conducted using Stata Statistical Software 14 (College Station, TX: StataCorp LP).

## Results

### Bacterial bloodstream infections

In this study, a total of 9,834 blood samples were processed. Study participants were 1 day to 80 years old. Median age of the study participants was 3 years (IQR: 1–10). 50.7% (*n* = 4,973) of the study participants were male. Of all blood culture samples, 14.3% (*n* = 1,410) showed bacterial growth including pathogens and contaminants such as skin flora and soil bacteria. From all blood cultures, 56 (0.6%) *S. aureus* were isolated. Study participants with *S. aureus*-positive blood cultures had a median age of 3 years (IQR 0–13 years) and 66.1% (*n* = 37) were male.

### Antimicrobial susceptibility

All *S. aureus* were sensitive to gentamicin, ciprofloxacin, linezolid, teicoplanin, vancomycin, tigecycline, fosfomycin, fusidic acid, rifampicin and moxifloxacin (Table 1). Inducible clindamycin resistance was not detected in any of the isolates.

**Table 1:** Percentage antibiotic resistance in *Staphylococcus aureus isolates*

Drug	Number of resistant isolates (%) *N* = 56
Oxacillin^a^	1 (1.8)
Penicillin^a^	55 (98.2)
Ciprofloxacin^b^	0 (0)
Moxifloxacin^b^	0 (0)
Teicoplanin^c^	0 (0)
Vancomycin^c^	0 (0)
Clindamycin^d^	1 (1.8)
Erythromycin^d^	2 (3.6)
Fosfomycin^d^	0 (0)
Fusidic acid^d^	0 (0)
Gentamicin^d^	0 (0)
Linezolid^d^	0 (0)
Rifampicin^d^	0 (0)
Tetracycline^d^	32 (57.1)
Tigecycline^d^	0 (0)
Trimethoprim/Sulfamethoxazole^d^	26 (46.4)

^a^B-lactams

^b^quinolone/fluorquinolones

^c^glycopeptides

^d^individual other group

The highest rate of resistance was for penicillin (*n* = 55; 98.2%) followed by tetracycline (*n* = 32; 57.1%) and trimethoprim/sulfamethoxazole (*n* = 26; 46.4%). In total, 35.7% (*n* = 20) of *S. aureus* were MDR, of which one was confirmed *mecA*-positive MRSA. MDR strains were most commonly resistant to the antibiotics penicillin, tetracycline and trimethoprim/sulfamethoxazole (*n* = 18; 90%).

### *S. aureus* spa types and detection of PVL

Twenty-five different *spa* types were identified in the isolates (Table 2). The most prevalent were t355 (*n* = 11; 19.6%) followed by t314 (*n* = 8; 14.3%), t084 (*n* = 8; 14.3%) and t311 (*n* = 5; 8.9%). The most frequent sequence types (ST) were ST152 (*n* = 17; 32.1%), followed by ST121 (*n* = 14; 26.4%) and ST15 (*n* = 9; 17.0%). The prevalence of PVL-positive isolates among all *S. aureus* was 75% (*n* = 42). Notable is that all isolates belonging to ST121 (*n* = 14; 100%) and ST152 (*n* = 17; 100%) and 55.6% (*n* = 9) of ST15 were PVL-positive. The MRSA isolate was PVL-negative *spa* type t786.

**Table 2:** Bacterial population structure of invasive *Staphylococcus aureus* isolates

CC (n)	ST (n)	*spa* type (n)	*Repeat pattern (aligned)*	*lukS-PV*/*lukF-PV* [Frequency (%)]
CC152 (17)	ST152 (17)	t355 (11)	07-56-12-17-16 -16-33-31-57-12	17 (100.0)
		t15285 (2)	07-56-12-17-342-16-33-31-57-12	
		t1123 (1)	07-56-12-17-16 -16- -31-57-12	
		t1299 (1)	07-56-12-17-16 - -33-31-57-12	
		t15281 (1)	07-56-12-17-16 -16-33-31-21-12	
		t15283 (1)	07-56-12-17-16 -16-33-31-57-12-12-12	
CC121 (14)	ST121 (14)	t314 (8)	08- -17-23-18-17	14 (100.0)
		t2304 (3)	08- -17-23-24	
		t645 (1)	14-44-13-12-17-23-18-17	
		t1077 (1)	14-44- -12-17-23-18-17	
		t1114 (1)	14-44-13-17-23-1-17	
CC15 (9)	ST15 (9)	t084 (8)	07-23-12-34-34-12-12- -23-02-12-23	5 (55.6)
		t2339 (1)	26-23-12-34-34-12-12-12-23-02-12-23	
CC5 (6)	ST5 (5)	t311 (5)	26-23-17-34-20-17-12-17-16	1 (20.0)
	ST6 (1)	t701 (1)	11-10-21-17-34-24-34-22-25-25	0 (0.0)
CC1 (2)	ST1 (2)	t127 (1) t591 (1)	07-23- -21-16-34-33-13	1 (50.0)
			07-23-21-21-16-34-33-13	
CC30 (2)	ST30 (2)	t363 (1)	15-16-02-25-17-24	2 (100.0)
		t8072 (1)	15-16-02-25- -24	
CC88 (2)	ST88 (2)	t448 (1)	07-12-21-17-13-13-34- -33-34	1 (50.0)
		t786 (1)	07-12-21-17-13- -34-34-33-34	
CC8 (2)	ST8 (1)	t008 (1)	11-19-12-21-17-34-24-34-22-25	0 (0.0)
	unknown (1)	t15282 (1)	11-10- -21-10-34-24-34-22-25-25	
CC45 (1)	ST508 (1)	t861 (1)	08-16-02-16-34-34-13-17-34-16-13	0 (0.0)
CC80 (1)	ST80 (1)	t376 (1)	07-23-12-34-34-34-33-34	1 (100.0)

Abbreviation: *CC*: clonal complex, *ST*: sequence type

## Discussion

We found very high rates of resistance in particular to penicillin but also to tetracycline and trimethoprim/sulfamethoxazole. African *S. aureus* strains from clinical infections are known to display high rates of resistance to the above-mentioned antibiotics but are also characterised by low resistance to other antibiotics indicated to treat infections with gram-positive bacteria [19–21]. This might reflect the frequent and repeated administration of locally available antibiotics, thus selecting for resistance and resulting in high frequencies of MDR. Our findings were in line with another study conducted in Ghana, where 32.1% of *S. aureus* isolated among different clinical specimens, including blood cultures, from urban and semi-urban hospitals, were reported being MDR [3]. In another Ghanaian study by Egyir et al., high proportions of *S. aureus* resistant to penicillin and tetracycline (97% and 42%, respectively) were seen in a collection of clinical samples, similar to what we observed in our study [3]. In contrast, the level of resistance for trimethoprim/sulfamethoxazole in our study was much higher (46%) as described in 2012 by Egyir et al. for urban and semi-urban Ghana (4%) [3] and more than twice as high as was described by Breurec et al. for African urban areas in Morocco, Cameroon, Madagascar, Niger and Senegal [4]. This might be due to overuse of this antibiotic in the past years in this particular rural region of Ghana, where the choice of drugs is limited.

We found only one MRSA among the *S. aureus* isolates (2%), which was similarly low to what was described by Egyir et al. (3%), and much lower than what was reported across the African continent [17, 22]. A study involving five major African towns reports an overall MRSA prevalence of 15% in clinical samples [4], and an even higher prevalence has been described in a Nigerian study (20%) [22]. The low frequency of MRSA observed in our study may be due to reduced drug pressure in the rural area under observation that may result from low prescription of third generation cephalosporins as these drugs tend to be more expensive [23].

In general, *spa* typing showed high genetic diversity as shown by the recovery of 25 different *spa* types from the isolates tested. The single detected MRSA isolate belongs to the typical MRSA clone of sequence type ST88 (t786) predominantly found in East, Central and West Africa [21]. *Spa* types t355, t084, and t314 were among the four most common *spa* types identified and are known to circulate in Ghana [3, 24]. These *spa* types have also been described as typical African clones in other studies [17, 25, 26]. Indeed, the results confirm stable populations of clinical *S. aureus* isolates in sub-Saharan Africa.

With regard to PVL, Africa has been considered a PVL-endemic region with very high rates of PVL-producing *S. aureus* strains (17%–74%), in particular MSSA [12, 17]. This was also emphasized in our study with PVL at 75%, which is among the highest prevalence described so far. Studies highlight the association of certain sequence types with the production of PVL mainly involving ST15, ST121 and ST151 [12]. Even though no statistical analyses were carried out due to low numbers, we observed a trend confirming this association in our bacterial isolates.

## Conclusion

The study demonstrated a high frequency of PVL-positive and genetically diverse MSSA lineages isolated from blood cultures in rural Ghana. The strains were characterised by high antibiotic resistance to commonly available antibiotics and low rates of MRSA and antibiotic resistance to other drugs specifically indicated for *S. aureus* infections.

The acquisition of the *mecA* and *mecC* genes by PVL-positive MSSA and hence the spread of PVL-positive MRSA could present a considerable challenge in disease management and infection control in the near future.

## Abbreviations

APH: Agogo presbyterian hospital
BD: Becton dickinson
BNITM: Bernhard nocht institute for tropical medicine
BURP: Based upon repeat patterns
CC: Clonal complex
DZIF: German centre for infection research
EUCAST: European committee on antimicrobial susceptibility testing
IQR: Interquartile range
IVI: International vaccine institute
KCCR: Kumasi centre for collaborative research in tropical medicine
KNUST: Kwame nkrumah university of science and technology
MDR: Multidrug resistance
MRSA: Methicillin-resistant *S. aureus*
MSSA: Methicillin-sensitive *S. aureus*
PCR: Polymerase chain reaction
PVL: Panton-valentine leukocidin
SMH: St. Michael’s hospital
ST: Sequence type
UKE: University medical centre Hamburg-Eppendorf
